# Overexpression of *luxS* Cannot Increase Autoinducer-2 Production, Only Affect the Growth and Biofilm Formation in *Streptococcus suis*

**DOI:** 10.1155/2013/924276

**Published:** 2013-11-07

**Authors:** Yang Wang, Li Yi, Zhicheng Zhang, Hongjie Fan, Xiangchao Cheng, Chengping Lu

**Affiliations:** ^1^College of Animal Science and Technology, Henan University of Science and Technology, Luoyang 471003, China; ^2^Key Lab of Animal Bacteriology, Ministry of Agriculture, Nanjing Agricultural University, Nanjing 210095, China; ^3^OIE Reference Laboratory for Swine Streptococcosis, Nanjing 210095, China; ^4^Department of Life Science, Luoyang Normal University, Luoyang 471022, China; ^5^Department of Animal Science and Technology, Jinling Institution of Technology, Nanjing 210038, China

## Abstract

LuxS/AI-2 quorum sensing (QS) system involves the production of cell signaling molecules via *luxS*-based autoinducer-2 (AI-2). LuxS has been reported to plays critical roles in regulating various behaviors of bacteria. AI-2 is a byproduct of the catabolism of S-adenosylhomocysteine (SAH) performed by the LuxS and Pfs enzymes. In our previous study, the function of LuxS in AI-2 production was verified in *Streptococcus suis* (SS). Decreased levels of SS biofilm formation and host-cell adherence as well as an inability to produce AI-2 were observed in bacteria having a *luxS* mutant gene. In this study, the level of AI-2 activity exhibits a growth-phase dependence with a maximum in late exponential culture in SS. An SS strain that overexpressed *luxS* was constructed to comprehensively understand the function of AI-2. Overexpressed *luxS* was not able to increase the level of *pfs* expression and produce additional AI-2, and the bacteria were slower growing and produced only slightly more biofilm than the wild type. Thus, AI-2 production is not correlated with *luxS* transcription. *luxS* expression is constitutive, but the transcription of *pfs* is perhaps correlated with AI-2 production in SS.

## 1. Introduction 

Quorum sensing is a chemical communication system among bacteria [1], which has been confirmed to be involved in a wide variety of biological processes, such as bioluminescence, biofilm formation, adherence, and virulence [2]. Several types of QS systems have been described in various species of bacteria. This LuxS/AI-2 QS system involves the production of cell signaling molecules via *luxS*-based autoinducer-2 (AI-2). AI-2 synthesis is linked to the metabolism of S-adenosylmethionine. Methylation reactions frequently use S-adenosylmethionine as the methyl donor to generate S-adenosylhomocysteine (SAH) [3]. SAH is hydrolyzed to adenine and 4,5-dihydroxy-2,3-pentanedione (DPD) by the nucleosidase Pfs and the LuxS enzyme [4]. Upon formation, DPD spontaneously cyclizes to form at least two different interspecies communication molecules described as AI-2 [5]. A broad range of gram-positive and gram-negative bacteria have been suggested to harbor *luxS* orthologs, most of which can produce AI-2 [3, 6]. AI-2 has been shown to be involved in biofilm formation and gene expression in many bacterial species. 

*Streptococcus suis* (SS) is a zoonotic pathogen associated with a wide range of diseases in pigs, including meningitis, septicaemia, pneumonia, endocarditis, and arthritis. It is also a problematic zoonotic agent for humans exposed to diseased pigs or their products because it causes life threatening diseases [7]. In a previous study, we found that the *luxS* gene of SS functions in AI-2 production, and mutant SS having a *luxS* deletion showed decreased biofilm formation, less adherence, and reduced virulence [8, 9]. 

The purpose of this study was to determine the regulation of AI-2 production and whether AI-2 production was regulated at the expression level of *luxS* gene whose product is directly involved in AI-2 generation, especially in the overexpression *luxS* SS strain. The results demonstrated that the level of AI-2 activity exhibits a growth-phase dependence, with a maximum in the late exponential culture in SS. Overexpressed *luxS* was not able to increase the level of *pfs* expression and produce additional AI-2, and therefore there was slower growth and a slight increase in biofilm formation compared to wild type.

## 2. Materials and Methods

### 2.1. Bacterial Strains, Culture Conditions, and Plasmids

Bacterial strains, plasmids, and growth conditions used are listed in Table 1. The HA9801 strain was isolated from SS2 infected pigs in the Jiangsu Province in 1998 and was confirmed as a virulent strain [10]. The *luxS* mutant of HA9801 (Δ*luxS*) and the complementation strain (CΔ*luxS*) were constructed in a previous study [8]. SS2 strains were grown in Todd-Hewitt broth (THB) (Difco Laboratories, Detroit, MI) medium or plated on THB agar with 5% (vol/vol) sheep blood. THB medium supplemented with 1% fibrinogen was used in the biofilm assay. When necessary, 100 *μ*g/mL of spectinomycin (Spc) (Sigma) or 4 *μ*g/mL of chloromycetin (Cm) (Sigma) were used for SS transformants, and 50 *μ*g/mL of ampicillin (Amp) (Sigma) was applied to screen the *E. coli* transformants. For overexpression plasmid construction, the structural gene of the *luxS* gene, including its own promoter, was amplified from chromosomal DNA of HA9801 by PCR using the primers Cup and Cdown (Table 1). The PCR product was cut with *EcoR* I/*BamH* I and ligated into the *EcoR* I/*BamH* I digested *E. coli/Streptococcus* shuttle vector pSET2 to generate the recombinant plasmid pSET2-C. Then, the recombinant plasmid and original pSET2 were separately electrotransformed into the parent strain competent cells. Transformed cells were selected with spectinomycin in THB medium. The transformants of SS containing the recombinant plasmid were designated as the overexpression strain of HA9801 (*luxS*+).

### 2.2. Real-Time PCR to Detect the Expression Level of *luxS* and *pfs* in the HA9801 and *luxS*+ Strains

Total RNA from growing in THB media at 1, 5, 10, and 14 h were extracted using Trizol reagent (Invitrogen), according to the manufacturer's protocol. SYBR Premix Ex Taq (TAKARA) was used for real-time PCR experiments in an ABI PRISM 7300 fast real-time PCR. The 16S rRNA gene was a housekeeping internal control [12]. The specific primers used for the various RT-PCR assays are listed in Table 1. At the end of each cycle, the fluorescence emitted by SYBR Green was measured. The comparative cycle threshold method (2^−ΔΔCT^ method) was used to analyze the mRNA levels [8].

### 2.3. AI-2 Bioassay

The AI-2 bioassay was performed according to the method previously described [8, 11]. The *Vibrio harveyi* BB170 was grown at 30°C in AB medium. After 4 h growth, *V. harveyi* BB170 cultures were diluted 1 : 5000 in fresh AB medium. 10 *μ*L of cell-free supernatant was added to 90 *μ*L of thawed diluted BB170 culture in a white, flat-bottomed, 96-well plate. Bioluminescence relative to uninoculated medium was calculated as fold induction [8]. For a single experiment, the *V. harveyi* bioassay was performed at least in duplicate for each sample. Experiments were repeated at least three times.

### 2.4. Growth Characteristics and AI-2 Activity in Different Growth Phases

The wild-type strain HA9801 and overexpression strain *luxS*+ were inoculated in THB media at the same concentration and cultured at 37°C. The optical density at 600 nm (OD600) of the culture was determined every hour to generate the growth curve.

The presence of AI-2 activity in the culture supernatant was tested every two hours as described above. The experiments were done in triplicate.

### 2.5. Biofilm Plate Assay

SS was tested for production of biofilm using the protocol described in our previous report [8]. Briefly, an overnight culture of SS was diluted to obtain an OD600 of 0.2 into fresh medium and incubated at 37°C for 24 h or 48 h before being stained with crystal violet. After fixing with methanol, staining was measured at 595 nm. All assays were performed in triplicate and repeated 3 times.

### 2.6. Statistical Analyses

Statistical analyses were carried out using the Graphpad Software package (GraphPad Software, La Jolla, CA). One way ANOVA was used in analysis of the biofilm formation. The mean values are shown in the figures. Where appropriate, the data were analyzed using the Student's *t* test, and a value of *P* < 0.05 was considered significant.

## 3. Results and Discussion

### 3.1. Transcriptional Analysis of the Level of *luxS* and *pfs* mRNA at the *luxS*+ and HA9801 Strains

**Figure 1:**
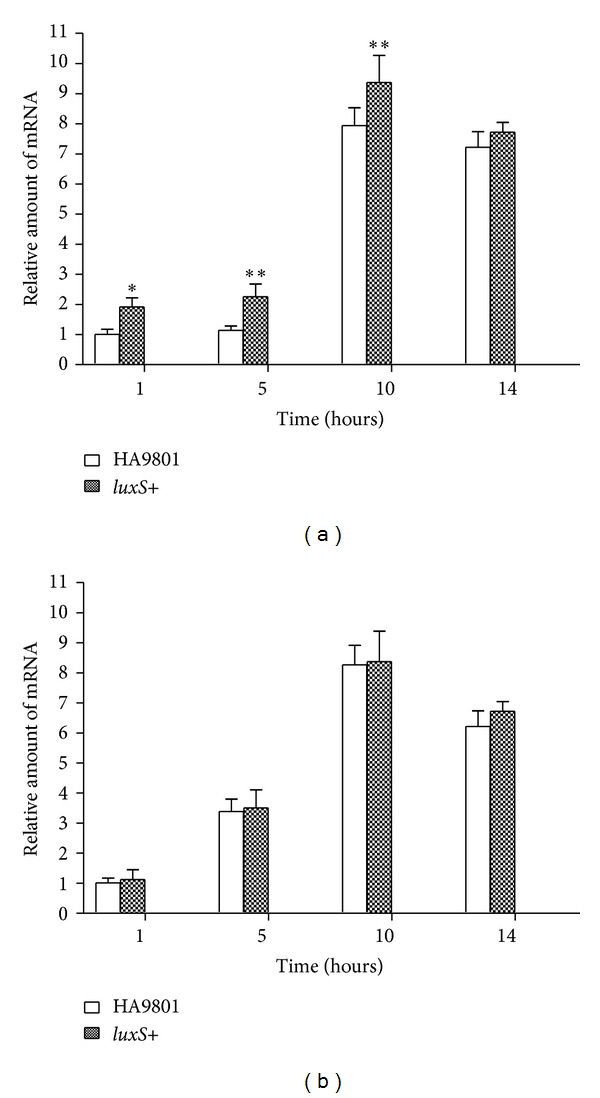
Analysis of the profile of* luxS* and *pfs *transcription in the different time. The expression level of *luxS* (a) and* pfs* (b) mRNA were analyzed using real-time PCR. Using 16S rRNA gene as reference gene, the expression level of 1 h is considered as 100%; relative expression is count as CT (2^−ΔΔCT^). Values are the means of results of three independent experiments. Error bars indicate standard deviations (*P* < 0.05).

Using overexpression techniques and knockout genes is the best way to study the *luxS* gene in *S. suis* HA9801. The expression level of *luxS* is displayed in Figure 1(a). The *luxS*+ and HA9801 strains have similar curves describing their respective *luxS* mRNA levels. The *luxS*+ strain had a higher *luxS* expression level than the HA9801 strain in all growth periods (*P* < 0.05, except at 14 h). In both *luxS*+ and HA9801, the level of *luxS* mRNA increased slowly from the 1 h to the 5 h, and then increased from the 5 h to the 10 h. After this, the level of *luxS* mRNA decreased slowly. The level of *luxS* was higher in the late exponential phase (10 h) than in the early exponential phase (5 h) and decline phase (14 h).

Figure 1(b) shows that the level of *pfs* mRNA quickly increased from 1 h to 10 h, reached a maximum level at the late exponential phase (10 h) in both the *luxS*+ and HA9801 strains, and then quickly decreased when SS was grown to the stationary phase (14 h). The *pfs* expression level of the *luxS*+ strain had similar curves with the HA9801 strain in all growth periods (*P* > 0.05). Overexpressing *luxS* in the HA9801 strain had no effect on the *pfs *expression level.

### 3.2. Growth-Dependent AI-2 Production by the *luxS*+ and HA9801 Strains

**Figure 2:**
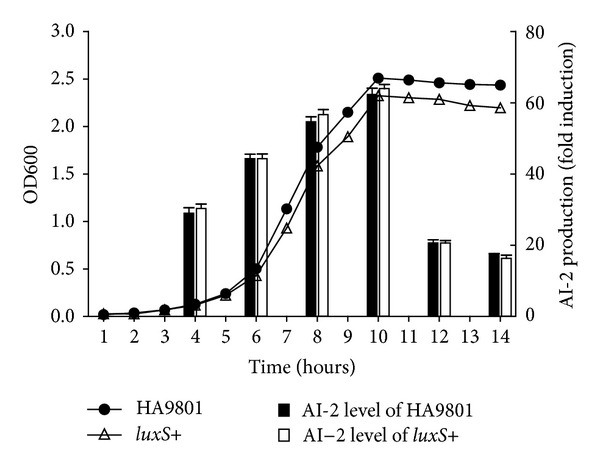
Growth-dependent AI-2 production by HA9801 and *luxS*+. The HA9801 and *luxS*+ were inoculated to THB media and cultured at 37°C. The OD600 of the culture was determined every hour to generate the growth curve. The ● and Δ indicate the parental and the *luxS*+ strains, respectively. Statistical analysis showed that *P* < 0.05 at 7–14 h, indicating that the *luxS*+ strain exhibited lagged growth relative to the WT strain. At various time points, AI-2 activity (left axis; column) is expressed as the fold induction of relative light units by comparing the level of luminescence to that induced by sterile medium. Values are means from three independent experiments.

In order to determine whether SS synthesis of AI-2 was growth-phase dependent, cell-free supernatants were collected at 4, 6, 8, 10, 12, and 14 h from a culture of SS HA9801 or *luxS+* (Figure 2). Prepared supernatants were stored at 4°C until the end of all experimental time courses, and all samples from each culture were assayed together in separate 96-well microtiter plates. The AI-2 activity increased over the first 10 h following inoculation. AI-2 activity peaked in the late exponential growth phase and then declined gradually in the stationary phase (Figure 2). The level of AI-2 activity exhibited a growth-phase dependence with a maximum in late exponential culture, as previously observed in *Bacillus anthracis* and *Streptococcus gordonii* [13, 14], suggesting that the activity of the molecule is density dependent in SS. Interestingly, the *luxS*+ strain exhibited lagged growth relative to the WT strain. Furthermore, when AI-2 activity of the *luxS*+ strain was compared with that of the WT strain, no significant difference was detected (*P* > .05). Our result that the strain with the plasmid-born *luxS* showed slower growth is in agreement with the observations of Van et al. for* Serratia plymuthica *RVH1 [15] and Zhang et al. for *Edwardsiella tarda* [16]. However, they all reported that overexpression of the *luxS* gene, which was accompanied by an increased production of AI-2, resulted in a slower growth [15, 16]. In contrast, these groups had not detected the level of *pfs* transcription in the overexpression strain. Perhaps the reason for this was that the overexpression strain with *luxS *on high-copy plasmid increased the level of *pfs* transcription and was a result of overproduction of AI-2. In our study, the overexpression of *luxS* did not seem to increase the level of AI-2 production, instead only affecting bacterial growth in SS.

### 3.3. AI-2 Activity in Different SS Strains

**Figure 3:**
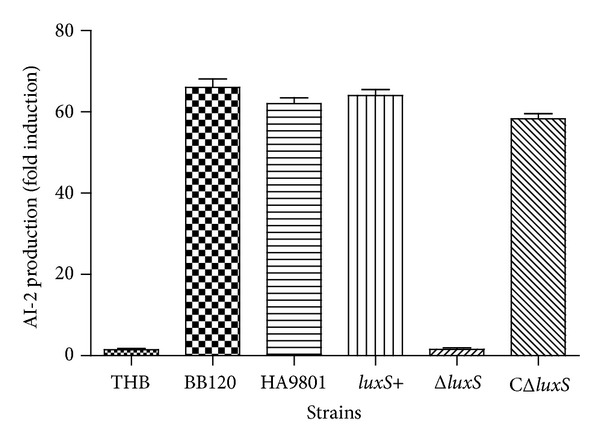
Production of AI-2 activity of the different strains. AI-2 activity of cell-free culture fluids in the stationary exponential phase was measured using the *V. harveyi* bioluminescence assay. AI-2 activity determination is expressed as relative light units and compared with the level of luminescence produced by the positive control (*V. harveyi* BB120). AI-2 activity of *V. harveyi* BB120 was set to 100% activity for normalization. *V. harveyi* BB120 served as a positive control and sterile THB medium as a negative control. HA9801, Δ*luxS*, CΔ*luxS*, and *luxS*+ refer to the WT strain, the *luxS *mutant strain, the complementation strain, and the overexpression strain. Values are means from three independent experiments. Error bars indicate standard deviations (*P* < 0.05).

To analyze the effect of AI-2 signaling in SS, we created a Δ*luxS* mutant strain, a complementation strain, and an overexpression strain (*luxS*+) in SS HA9801. In the late exponential to the stationary exponential phase, the SS culture supernatant had the largest AI-2 luminescence response (Figure 2). The culture supernatant from the Δ*luxS* mutant strain induced a luminescence signal that was comparable to the signal from the negative control, which was much lower than that of the WT strain. However, the complementation strain restored AI-2 to the level of the WT strain. The overexpression strain (*luxS*+) generated similar levels of bioluminescence as the WT strain HA9801 (Figure 3).

These results suggest that in SS, *luxS* is necessary for AI-2 synthesis and encodes a functional AI-2 or AI-2-like molecule. However, the *luxS*+ overexpression strain did not produce a higher level of AI-2 than was produced by the WT strain, possibly because *luxS* transcription does not correlate with AI-2 production; however, transcription of *pfs* is highly correlated with AI-2 production in SS. Similar results have been reported by Beeston that *luxS *expression is constitutive but that the transcription of *pfs* is tightly correlated to AI-2 production in *Salmonella serovar Typhimurium* [17]. Consequently, it follows that the level of *pfs* transcription was not altered in the *luxS*+ strain (Figure 1(b)).

### 3.4. The Ability of Different SS Strains to Form Biofilm

**Figure 4:**
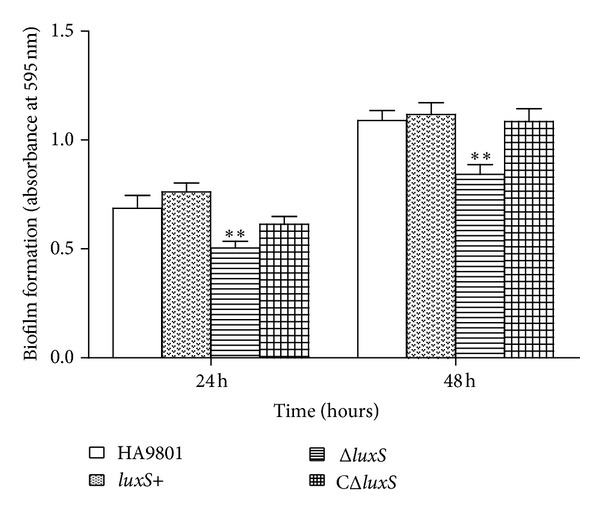
Quantitative determination of biofilm formation of the different strains. All strains were cultured in THB medium supplemented with 1% fibrinogen. Each strain was tested in 8-wells of a 96-well microtiter plate. Time course of biofilm formation of the different strains for 24 h or 48 h. HA9801, Δ*luxS*, CΔ*luxS, *and *luxS*+ refer to the WT strain, the *luxS *mutant strain, the complementation strain, and the overexpression strain. The biofilm formation by the Δ*luxS* was significantly decreased compared to that of the WT strain (*P* < 0.01). The *luxS*+ overexpression strain was slightly increased to those of the WT strain and CΔ*luxS.* Negative control (NC) wells contained broth only. The columns represent the means and standard deviations of four or more experiments. The asterisk showed significant difference (*P* < 0.05).

The abilities of the wild type, mutant, complementation, and overexpression strains to develop biofilms in 96-well microtiter plates were determined using the Crystal Violet (CV) assay (Figure 4). When these plates were incubated under the same culture conditions at 37°C for 24 h without agitation, the WT strain HA9801 formed considerably more biomass (OD_595_, 0.69 ± 0.11) than the *luxS *mutant strain (OD_595_, 0.50 ± 0.08) (*P* < 0.01). Furthermore, the biofilm formed by the *luxS*+ overexpression strain (OD_595_, 0.76 ± 0.12) was slightly increased to those of the WT strain HA9801 and the complementation strain (Figure 4).

Because the *luxS* gene plays a vital role in biofilm formation, adherence, and gene expression in SS [8], it can be hypothesized that the overexpression level of *luxS* mRNA can increase SS biofilm formationviaregulation of the major adhesion-associated genes such as* gapdh* and* fbps*, which mediated the adhesion, invasion, and colonization capacity of SS to host cells [18]. The results are consistent with those from qRT-PCR, in which expression of *gapdh* and* fbps* was slightly increased in the* luxS*+ (date not shown). After 48 h of plate incubation, all tested strains showed a significant increase in biofilm formation compared to plates incubated for 24 h (*P* < 0.05). Similar results have been reported for *Bacillus cereus *[19]. In one study, Rickard reported that the biovolume of a monospecies biofilm increased over time following inoculation [20]. Time-resolved inspection of biofilm development revealed that each strain exhibited an increase in total biovolume over time. 

## 4. Conclusions

This study has demonstrated that the level of AI-2 activity exhibits a growth-phase dependence with a maximum in late exponential culture in SS, and overexpressed *luxS* is not able to increase the level of *pfs* expression and produce additional AI-2, and therefore has slower growth and slightly increases biofilm formation. Thus, AI-2 production is not correlated with *luxS* transcription. *luxS* expression is constitutive but, the transcription of *pfs *is perhaps correlated to AI-2 production in SS.

## Figures and Tables

**Table 1:** Characteristics of bacterial strains, plasmids, and primers used in this study.

Strain, plasmid, and primer	Relevant characteristics^a^ or sequence (5′-3′)^b^	Source of references
Strains		
HA9801	Virulent strain of SS2 isolated from dead pig	Collected in our laboratory
Δ*luxS *	Mutation in *luxS* gene of HA9801; Cm^r^	[8]
CΔ*luxS *	Complemented strain of Δ*luxS*; Spc^r^; Cm^r^	[8]
*luxS*+	Overexpressing strain of *luxS;* Spc^r^	In this study
*E. coli *DH5a	Cloning host for maintaining the recombinant plasmids	Invitrogen
*V. harveyi *BB170	BB120 luxN::Tn5 (sensor 1^−^, sensor 2^+^) *V. harveyi *	[11]
*V. harveyi* BB120	Wild type *V. harveyi *	[11]
Plasmid		
pMD18-T vector	Clone vector	Takara
pSET2 vector	*E. coli-S. suis* shuttle vector; Spc^r^	[8]
Primers		
Cup	CCGG AATTCACCTCGGTTCCTTGTCTG	In this study
Cdown	GCGGGATCCGCTTCTTGTTCTGCGTTT; amplifies the structural gene of the *luxS *gene, including its own promoter (922bp)	In this study
LuxS-F	CAAGTCATCAAGTCGAGTTTGGAAG	In this study
LuxS-R	TCTTTAGCTGAATGAAGGCTGTGG; real-time for *luxS *	In this study
Pfs-F	CGTTCTTGTTCAGTCAGGTATCGG	In this study
Pfs-R	GATGGCAATACCTTCAGCAACAG; real-time for *pfs *	In this study
16S rRNA-F	GTTGCGAACGGGTGAGTAA	In this study
16S rRNA-R	TCTCAGGTCGGCTATGTATCG; real-time for 16S rRNA	In this study

^a^Ap^r^, ampicillin resistant; Spc^r^, spectinomycin resistant; Cm^r^, chloramphenicol resistant; ^b^Underlined nucleotides are restriction sites of the enzymes.
